# A follow-up analysis of positron emission tomography/computed tomography in detecting hidden malignancies at the time of diagnosis of membranous nephropathy

**DOI:** 10.18632/oncotarget.7506

**Published:** 2016-02-19

**Authors:** Zhonglin Feng, Shuxia Wang, Yanlin Huang, Xinling Liang, Wei Shi, Bin Zhang

**Affiliations:** ^1^ Department of Nephrology, Guangdong General Hospital, Guangdong Academy of Medical Sciences, Guangzhou, China; ^2^ Department of Nuclear Medicine and PET Center, Guangdong General Hospital, Guangdong Academy of Medical Sciences, Guangzhou, China; ^3^ Medical Genetics Center of Guangdong Women and Children Hospital, Guangzhou, China

**Keywords:** membranous nephropathy, malignancy, PET/CT, Pathology Section

## Abstract

Membranous nephropathy (MN) is the most common kidney disease reported in a variety of malignant diseases. Search for an occult malignancy in MN has presented special challenges. 124 MN patients with a physical examination not suspicious for cancer underwent screening for an occult malignancy with either 18F-Fluorodeoxyglucose positron emission tomography/computed tomography (FDG-PET/CT) scanning (*n* = 49) or conventional screening (*n* = 75) at the time of diagnosis of MN, and were followed up (median,28 months). 154 patients who refused to undergo any screening were followed up (median, 30 months). In FDG-PET/CT cohort, 5 (10.20%) patients were screened and confirmed as malignancy, in contrast, 1 (1.33%) patient in conventional screening cohort. During follow-up, none of malignancy was detected in FDG-PET/CT cohort, 3(4.05%) patients in conventional screening cohort, and 8(5.19%) patients in no-screening cohort. All 6 cases of cancer were detected at early stages and underwent curative resection, and after the resection, proteinuria decreased. In contrast, 11 cases of cancer detected during follow-up died without any remission of proteinuria. These preliminary data provide the first evidence for a potential cancer surveillance that the malignancy screening either through conventional or by PET-CT at the diagnosis of MN led to an early diagnosis and curative treatment.

## INTRODUCTION

Membranous nephropathy (MN) is the most common kidney disease reported in a variety of malignant diseases. The list of malignancies occurring excessively in MN has expanded to include a much broader range of neoplasms, including carcinomas of lung, esophagus, colon, breast, stomach, prostate and lymphoma etc [1, 2]. MN is the most common cause of nephrotic syndrome in adult. It is defined at the histopathologic level by the presence of immune complexes on the extracapillary side of the glomerular basement membrane. The exact etiology of MN is still unclear. Approximately 75% of the cases of MN are idiopathic, or primary, membranous nephropathy (IMN). The remainder is associated with a variety of conditions thought to secondarily cause MN; these include systemic lupus erythematosus, hepatitis B antigenemia or other chronic infections, and, historically, a number of drugs and toxins such as therapeutic gold salts, D-penicillamine, and agents containing mercury [3].

The association between MN and malignancy was first reported in 1966, when Lee et al.[4] reported that 11% of patients with nephrotic syndrome had carcinoma. Since then, several case series suggested a link between MN and malignancy; although the true prevalence of malignancy in patients with MN remains unknown, it has been variously estimated as ranging between 5 and 22% [5-10]. In the largest such study, Lefaucheur et al.[10] reported a prevalence of malignancy of 10% in a review of 240 patients with biopsy-proven MN, and this was about 10-fold higher in patients with MN than in the general population. Recently, a meta-analysis of cohort studies in Caucasian population revealed a close association between MN and cancer with a prevalence rate of 10% and emphasized the importance of an extensive screening for malignancy in patients diagnosed with MN [11].

Search for an occult malignancy in patients with newly diagnosed MN has presented special challenges. The evidence review in the Kidney Disease: Improving Global Outcomes (KDIGO) Clinical Practice Guidelines (http://www.kdigo.org) has confirmed the paucity of data to support the recommendations for screening an occult malignancy in MN, and further studies are needed to investigate the effectiveness of screening modality in this population [12]. In MN, malignancies can occur in any anatomic site, making it difficult to identify practical, effective screening and prevention strategies [11]. On the other hand, of the patients with malignancy-associated MN, only 20±6.8% had the diagnosis of malignancy before the diagnosis of MN. For the remaining 80±15%, the cancer was diagnosed at the time of or following the diagnosis of MN [11]. MN may be the first clinical manifestation of an occult malignancy, and patients suffering from MN develop diverse spectrum of new cancers during the following years [9-11]. Therefore, screening at the time of diagnosis of MN is worthwhile, as this may be the first sign of occult malignancy. Of these with malignancy-associated MN, only a minority of patients were known to have symptoms related to their cancer at the time of kidney biopsy. In a majority of the cases, the malignancy was asymptomatic and only recognized by systematic diagnostic procedures triggered by the diagnosis of MN [10].

It is reasonable to perform routine screening for malignancy in patients with newly diagnosed MN once other secondary causes have been excluded. The conventional screening modality may include a complete physical examination, laboratory tests, gastrointestinal endoscopy, ultrasonography, X radiation study, tumor markers and gynecologic examination in women. However, data from these conventional screening modalities in MN is still unavailable. On the other hand, ^18^F-Fluorodeoxyglucose positron emission tomography/computed tomography (FDG-PET/CT) scanning can detect subclinical primary tumors as well as metastatic lesions in a wide range of malignancies [13]. FDG-PET/CT is more accurate in detecting cancer and provides fewer equivocal findings than PET alone [14-17], CT alone, or separately acquired PET and CT studies in a head to head comparison [18]. For cancer staging, FDG-PET/CT is also more accurate than either modality alone, as shown for lung cancer [19,20], colorectal cancer [17], and lymphoma [21].

In this study, we explored the use of FDG-PET/CT scanning for early detection in patients with a physical examination not suspicious for cancer at the time of diagnosis of MN, and compare the effectiveness of FDG-PET/CT scanning with that of routine conventional screening.

## RESULTS

**Figure 1:**
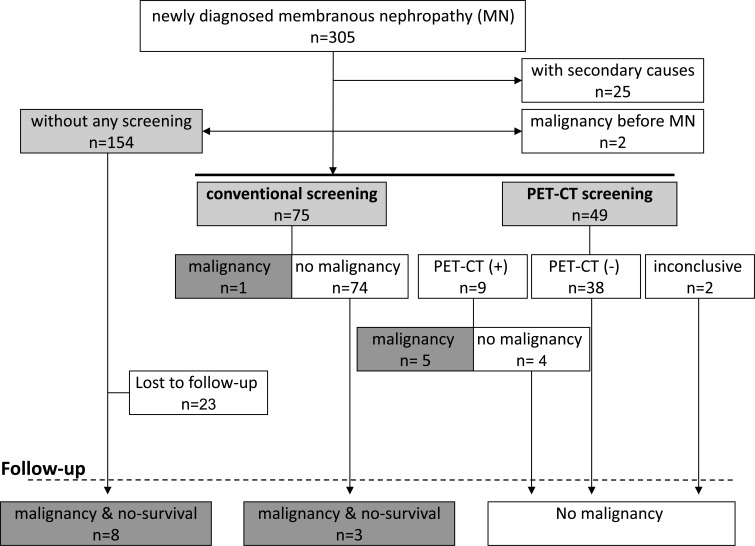
FDG-PET/CT and conventional malignancy screening PET/CT ^18^F-Fluorodeoxyglucose positron emission tomography/computed tomography.

Between November 2007 and April 2014, 305 consecutive patients were newly diagnosed with MN (Figure 1). Of these,124 presumed idiopathic MN patients with a physical examination not suspicious for cancer underwent screening for an occult malignancy with either FDGPET/CT scanning (*n* = 49) or the conventional screening (*n* = 75) (Figure 1). Of these remaining 154 MN patients, they refused to received any screening. The baseline characteristics of 3 cohorts were similar (Table 1).

**Table 1:** Characteristics of study cohorts

	no screening	conventional screening	PET-CT screening	*p* value
	*n* = 154	*n* = 75	*n* = 49	
age	50(26.25)	56(14.75)	59.5(19.75)	0.064
female sex, n (%)	91(59.1)	40(53.33)	24(48.98)	0.410
proteinuria (g/24 h)	2.92(3.44)	3.57(4.51)	4(5.42)	0.089
eGFR (ml/min*1.73m2)	89.4(42.99)	73.45(46.53)	72.53(56.16)	0.643
smoking, n (%)	11(7.14)	5(6.67)	4(8.16)	0.951
alcohol abuse, n (%)	3(1.95)	3(4)	2(4.08)	0.548
hypertension, n (%)	36(23.38)	14(18.67)	8(16.33)	0.492
malignancy total, n (%)	8(5.19)	4(5.41)	5(10.42)	0.409
malignancy at kidney biopsy, n (%)	---	1(1.33)	5(10.20)	0.035
malignancy during follow-up, n (%)	8(5.19)	3(4.05)	0	0.272
follow-up time, months	30 (36.98)	29(34.75)	27(15)	0.582
lost to follow-up, n (%)	23(14.94)	3(4)	1(2.04)	0.005

Of the 49 patients, FDGPET/CT scanning detected likely malignant lesions in 9 (18.37%), among which 5 (10.20%) patients were subsequently confirmed as malignant (Figure 1). In contrast, of the 75 patients, the conventional screening detected malignant lesion in 1 (1.33%).

Three (4.05%) patients who were initially negative in the conventional screening were subsequently confirmed as malignant (Figure 1 and Tables 1 & 2) during a median follow-up of 29 months (IQR, 34.75 months). In contrast, during a median follow-up of 27 months (IQR, 15 months), none of the patients with initially negative FDGPET/CT was identified as being malignant. As a result, the sensitivity of FDG-PET/CT was 100% (5/5), while that of the extensive conventional screening was only 25% (1/4). In contrast, of these 154 patients who refused to received any screening, 8(5.19%) patients were subsequently confirmed as malignant (Figure 1 and Table 1 & 2) during a median follow-up of 30 months (IQR, 36.98 months). Finally, a total of 5.41% patients were identified as having cancer in the conventional screening cohort, and 5.19% in the no-screening cohort, which were lower than 10.42% in the FDG-PET/CT scanning cohort (Figure 1 and Tables 1 & 2).

**Table 2:** MN Patients with finally confirmed malignancies

	age/gender	screening	malignancy	pathology/stage	follow-up (mo)	survival	surgical resection	proteinuria (g/24 h)	proteinuria after resection*
1	49/M	Conventional(+)	colon	adenocarcinoma/T3N2M1	74	yes	yes	3.14	disappearance
2	73/M	PET-CT(+)	prostate	adenocarcinoma/Gleason score 4+3	35	yes	yes	2.87	reduction
3	65/M	PET-CT(+)	lung	adenocarcinoma/T1N0M0	35	yes	yes	15.43	reduction
4	50/F	PET-CT(+)	thyroid	adenocarcinoma/T1N0M0	28	yes	yes	5.25	reduction
5	78/M	PET-CT(+)	lung	adenocarcinoma/T1N0M0	46	yes	yes	1.51	disappearance
6	69/M	PET-CT(+)	stomach	adenocarcinoma/T1N1M0	81	yes	yes	8.15	disappearance
7	75/F	Conventional(−)	breast	N/A	28	no	no	4.56	no remission
8	54/M	Conventional(−)	colon	N/A	36	no	no	1.40	no remission
9	74/M	Conventional(−)	lung	N/A	51	no	no	3.59	no remission
10	69/F	no	lung	N/A	15	no	no	3.95	no remission
11	66/M	no	stomach	N/A	19	no	no	4.46	no remission
12	64/F	no	lung	N/A	32	no	no	3.9	no remission
13	31/M	no	lung	adenocarcinoma/T2N1M1	18	no	yes	1.62	no remission
14	76/M	no	colon	N/A	73	no	no	2.88	no remission
15	74/F	no	N/A	N/A	77	no	no	2.14	no remission
16	74/M	no	N/A	N/A	58	no	no	7.4	no remission
17	89/M	no	prostate	N/A	58	no	no	1.84	no remission

* Proteinuria after resection of malignancy: 1,disappearance: proteinuria<0.15g/day; 2, reduction: proteinuria <1 g/day.

Of the total 17 patients with confirmed malignancies in 3 cohorts, tumors were found to occur in diverse anatomic sites, including lung (6), colon (3), prostate (2), stomach (2), breast (1) and thyroid (1) (Table 2). Of these, six patients underwent either FDGPET/CT scanning (*n* = 5) or the conventional screening (*n* = 1) at the time of diagnosis of MN. All these six patients were diagnosed with adenocarcinoma at early stages and underwent curative resection, and after surgical resection the amount of proteinuria decreased (Table 2). Unfortunately, 3 patients who were initially negative in the conventional screening cohort and 8 patients in the no-screening cohort developed a malignancy during the follow-up. All these 11 patients died either as a direct or indirect result of their tumor and had persistent proteinuria without any remission(Table 2). Patients with malignancy-associated MN were significantly older than patients without malignancies, and had lower female to male sex ratio and lower estimated glomerular filtration rate (Table 3). However, these differences were no longer significant after adjusting for age.

**Table 3:** Characteristics of MN patients with malignancy or without malignancy

	malignancy	no-malignancy	*p* value	Age-adjusted *p* value
	*n* = 17	*n* = 261		
age	69(15.5)	52(25)	<0.001	
female sex, n (%)	5(29.41)	150(57.47)	0.024	
proteinuria (g/24 h)	3.59(2.92)	3.28(4.38)	0.637	0.785
eGFR (ml/min*1.73m2)	55.06(58.87)	83.18(52.81)	0.009	0.592
smoking, n (%)	1(5.88)	19(7.28)	>0.999	0.421
alcohol abuse, n (%)	0	8(3.07)	>0.999	0.998
hypertension, n (%)	3(17.65)	55(21.07)	0.977	0.156

## DISCUSSION

In clinical practice, search for an occult malignancy in patients with MN has presented special challenges because of the diverse spectrum of MN-associated malignancies [1, 2]. The KDIGO Clinical Practice Guideline for Glomerulonephritis (http://www.kdigo.org) has confirmed the paucity of data for screening an underlying malignancy in this population. This follow-up analysis of cohort study showed that the malignancy screening either through conventional or by FDG-PET/CT scanning at the time of MN diagnosis led to an early diagnosis of cancer and curative treatment. It may be helpful for the design of prospective randomized studies.

Our results showed that whole-body FDG-PET/CT scanning at the time of diagnosis of MN identified occult malignancies in 10.2% of patients, which is similar with the previous reports [10,11]. In contrast, the conventional screening detected malignant lesion only in 1.33%. The FDG-PET/CT scanning is a non-invasive whole-body imaging technique routinely used for the diagnosis of malignancies with high sensitivity [22, 23]. The sensitivity of FDG-PET/CT was 100%, while that of the conventional screening was only 25%. In earlier studies, the majority of MN-associated malignancies were discovered at the time of or following the diagnosis of MN [11]. And in this study, of the total 17 patients with confirmed malignancies, tumors were found to occur in diverse anatomic sites, including lung (6), colon (3), prostate (2), stomach (2), breast (1) and thyroid (1). The result is in agreement with earlier series in which cancers in MN can occur in diverse anatomic sites, [7-9,24,25], making it difficult to identify practical, effective screening and prevention strategies. Although the rate of detection of cancer on conventional screening remained lower compared to the FDG-PET/CT scanning, the search of a hidden malignancy either through conventional or by FDG-PET/CT scanning at the time of MN diagnosis appears as an attractive strategy since early diagnosis could imply better outcomes[9-11]. In this study, all these 6 patients who underwent screening and finally confirmed as having cancer (FDG-PET/CT scanning, *n*= 5 or the conventional screening, *n* = 1) were showed to be at early stages and underwent curative resection, and after surgical resection, the amount of proteinuria decreased. However, 3 patients who were initially negative in the conventional screening cohort and 8 patients in the no-screening cohort developed a malignancy, and died either as a direct or indirect result of their tumor and had persistent proteinuria without any remission.

Our study has several limitations. First, this is a retrospective cohort study and there is no independent validation cohort, although it is the first large study assessing FDG-PET/CT for the screening of hidden cancer in patients with MN. Nevertheless, it may be useful for the design of new prospective randomized studies comparing conventional screening strategy with FDG-PET/CT screening, also focusing on impact on survival. Second, a drawback of the application of FDG-PET/CT for the search of a hidden malignancy is the high percentage of false-positive findings, leading to unnecessary additional explorations. According to our results, the positive predictive value (PPV) of FDG-PET/CT was only 55.6% (5/9). Considering the high cost of PET-CT, relatively low PPV for malignancy in MN, routine PET-CT screening in MN patients would be difficulty in current clinical practice. Thus stratify the MN patients for screening is particularly important. It is reported that anti-PLA2R is in an association with malignancy occurrence, and the status of anti-PLA2R may help stratify the MN patients for screening and justify the use of PET-CT screening [26, 27]. Third, as FDG-PET/CT is not widely available and interpretation depends heavily on expertise, the implementation of this screening strategy may be difficult.

## MATERIALS AND METHODS

### Patients

A total of 305 consecutive patients with biopsy proven MN between November 2007 and April 2014 were identified (Figure 1). The kidney biopsies were performed at Guangdong General Hospital, South China University of Technology (SCUT). Diagnostic features of MN included capillary wall thickening, normal cellularity, IgG and C3 along capillary walls on immunofluorescence, and subepithelial deposits on electron microscopy. Of these, 25 patients who had secondary causes were excluded by using history, physical exam, and appropriate laboratory tests (Figure 1). Two patients who had malignancies before the diagnosis of MN were also excluded. 154 patients refused to undergo any malignancy screening at the time of kidney biopsy. In the end, 124 patients with presumed idiopathic MN signed a fully informed consent to undergo the conventional screening (*n* = 75) or FDG-PET/CT scanning (*n* = 49) to investigate the presence of an occult malignancy at time of MN diagnosis (Figure 1).

This is a retrospective cohort study. The cohort of the conventional screening (*n* = 75) served as the control cohort and were not randomly assigned. But as shown in Table 1, baseline characteristics of both cohorts are comparable. The study protocol and data handling procedure approved by the Ethics Committee of Guangdong General Hospital.

### Conventional screening *versus* FDG-PET/CT scanning

The spectrum of MN-associated cancers was diverse and the majority of tumor-associated MN patients had no cancer-related to symptoms at kidney biopsy [1, 2]. Considering these concerns, we presumed that the whole-body PET-CT could be more suitable to detect such a broad spectrum of hidden cancers than the conventional screening, even if the conventional malignancy screening were extensive.

Conventional malignancy screening in this study included a complete physical examination, laboratory tests (complete blood count, fecal occult blood testing and serum chemistry panel), upper gastrointestinal endoscopy, colonoscopy, ultrasonography of thyroid gland and abdominal organs, or thoracoabdominal X radiation, tumor markers (CA125, CA199, carcinoembryonic antigen, prostate-specific antigen), and gynecologic examination in women, including ultrasonography.

FDG-PET/CT scanning was performed using an integrated whole body FDG-PET/CT scanner (Biograph Duo LSO, Siemens). ^18^F-FDG was synthesized with an automatic chemical module and an on-site cyclotron (RDS111, CTI Inc., Chicago, Illinois, USA). Radiochemical purity was greater than 95%. Patients were fasted for at least 4hr before injection, serum glucose was ≤150 mg/dL. 5.92MBq/kg ^18^F-FDG was administered intravenously through iv canular, and uptake time was 60 min+10min, 3D requisition, 2 min/bed, iterative reconstruction (iteration 4, subsets 8). CT part of PET/CT was performed without breath hold (120 kVp, 50 mAs). Two double certified physicians closely monitored patients on-site, one is a nuclear medicine physician and the other is a radiologist. Image report was written by the two physicians, combining PET and CT characteristics with consensus. In case of abnormal or suspicious findings, the patient was informed and appropriate diagnostic procedures to confirm or rule out malignancy were programmed, including biopsy, resection, or follow up. The follow up was made by telephone interview in a blinded manner.

### Statistical analysis

Qualitative data was expressed as percentages, and quantitative data was presented as median and 25-75% interquartile range or mean and standard deviation. The Fisher exact, Pearson chi-square test, continuity correction chi-square test or chi-square test were used to assess the relationships. The Mann-Whitney U or T test were used to assess the relationships between quantitative variables. Statistical significance was set at P less than 0.05, and all analyses were performed with the Statistical Package for the Social Sciences version 15.0 (SPSS Inc, Chicago, Ill).
